# Disaster declarations associated with bushfires, floods and storms in New South Wales, Australia between 2004 and 2014

**DOI:** 10.1038/srep36369

**Published:** 2016-11-07

**Authors:** T. Sewell, R. E. Stephens, D. Dominey-Howes, E. Bruce, S. Perkins-Kirkpatrick

**Affiliations:** 1 Asia – Pacific Natural Hazards and Disaster Risk Research Group, School of Geosciences, The University of Sydney, Sydney, NSW 2006, Australia; 2 Geocoastal Research Group, School of Geosciences, The University of Sydney, Sydney, NSW 2006, Australia; 3 Climate Change Research Centre and ARC Centre of Excellence for Climate System Science, University of New South Wales, Sydney, NSW 2052, Australia.

## Abstract

Australia regularly experiences disasters triggered by natural hazards and New South Wales (NSW) the most populous State is no exception. To date, no publically available spatial and temporal analyses of disaster declarations triggered by hazards (specifically, bushfires, floods and storms) in NSW have been undertaken and no studies have explored the relationship between disaster occurrence and socio-economic disadvantage. We source, collate and analyse data about bushfire, flood and storm disaster declarations between 2004 and 2014. Floods resulted in the most frequent type of disaster declaration. The greatest number of disaster declarations occurred in 2012–2013. Whilst no significant Spearman’s correlation exists between bushfire, flood and storm disaster declarations and the strength of the El Niño/Southern Oscillation (ENSO) phase, we observe that bushfire disaster declarations were much more common during El Niño, and flood disaster declarations were five times more common during La Niña phases. We identify a spatial cluster or ‘hot spot’ of disaster declarations in the northeast of the State that is also spatially coincident with 43% of the most socio-economically disadvantaged Local Government Areas in NSW. The results have implications for disaster risk management in the State.

Hazards and the disasters they may cause adversely affect societies and seriously disrupt socio-ecological systems. Australia is at risk from a range of hazards including (amongst others) bushfires, droughts, floods, heatwaves, storms of various types and tropical cyclones. Implicit here is the distinction between events labeled as hazards from those referred to as disasters. An actual disaster represents a complex combination of physical hazard processes, social vulnerability and response and adaptive capacity. Definitions of disasters have long emphasized this coupling of human and environmental systems^1^,^2^ and both environmental and human factors play a role in determining where disasters occur, and when. The spatial distribution of hazards depends on a combination of geomorphological, geophysical, meteorological and climatological factors^1^. Areas in the tropics will be more vulnerable to the occurrence of tropical cyclones, for example, and areas of low elevation near the coast will be more vulnerable than higher areas to storm surges. However, just as important are social factors such as population densities and distribution, vulnerability, resilience and adaptive capacity and emergency preplanning and management, which determine how many people will be impacted, and to what extent^1^,^3^.

In Australia, the most commonly occurring and costly type of declared disasters are associated with hydro-meteorological hazards. Since the turn of the millennium, Australia has been affected by hydro-meteorological hazards leading to declared disasters that have commanded significant national and international attention. They include for example, the Millennium Drought of 1995–2009; the Sydney hailstorm of 1999; Cyclone Larry in 2006; the Victorian bushfires of 2009; the Queensland floods in 2011 and the New South Wales bushfires of 2013. In 2009 alone, the Black Saturday Victorian bushfires and the New South Wales and Queensland storms and floods killed hundreds of people and caused $2 billion + of insured damage. Uninsured losses were even higher^4^. Despite the influences of natural variability and some scientific uncertainty, the frequency and/or intensity of different hydro-meteorological hazards are expected to vary, and in many cases, increase in the 21^st^ century as a consequence of anthropogenic climate change^5^,^6^,^7^,^8^.

The State of New South Wales (NSW) is located in southeast Australia (Fig. 1A). It comprises 800,642 km^2^ in area and ranges from arid, hot dry conditions in the west, to tropical wet conditions in the northeast, with temperate conditions along much of the coast and alpine cold conditions in the central southeast. The capital is Sydney and the State population stands at 7,250,000. NSW is a ‘coastal State’ with 75% of residents in the Greater Metropolitan Region. This is defined as all Local Government Areas or LGAs within the Sydney Statistical Division and the Newcastle and Wollongong Statistical Subdivisions (Fig. 1B). NSW is currently divided in to 152 LGAs and LGAs are the lowest administrative unit of government in Australia. NSW is regularly affected by hazards resulting in what the government terms *Natural Disaster Declarations*^9^ and more than 90% are hydro-meteorological related.

The NSW government defines a ‘natural disaster’ as *“a serious disruption to a community or region caused by the impact of a naturally occurring rapid onset event that threatens or causes death, injury or damage to property or the environment and which requires significant and coordinated multi-agency and community response”* (Emergency Management NSW, 2010, p.x)^10^.

In this definition, community disruption is mentioned first, prioritising human impacts above others such as property and environmental damage. This means that assessments of, and about disasters, are made in primarily human terms. Consequently, the designation and declaration of a hazard as a disaster is a socially constructed phenomena as opposed to an event that reaches or exceeds a specific objective magnitude or threshold. That is, not all hazards lead to disaster declarations – no matter how large those events are.

Under Federal and State legislation, the NSW government plans for disasters, triggered by natural and non-natural events. The NSW State Emergency Management Committee (SEMC), established under the *State Emergency and Rescue Management Act 1989 No 165* (SERM Act), ensures NSW has a system to cope with disasters. At the heart of the National Risk Assessment Guidelines that the NSW government and its emergency managers use to plan for disasters is the requirement for fundamental data on the types, frequencies, distributions and impacts of hazards^11^. Given the variety of hazards that may affect NSW, we note that there are no publically available spatial and temporal analyses of disaster declarations. This contrasts with other jurisdictions around the world such as in the United States where others have conducted similar spatial and temporal analyses of hazards and their accompanying disasters using publically available datasets^12^,^13^,^14^,^15^. Further, in NSW, no studies have explored the relationships, if any, between socio-economic disadvantage and the spatial occurrence of hazards that result in disaster declarations. This omission stands as an obstacle for supporting effective disaster risk management planning and policy.

Given the omissions just identified, the aims of this study are to:

1. source, collate, analyze and map information about disaster declarations in NSW between 2004 and 2014 focusing on the commonly recurring and sudden onset types of bushfires, floods and storms mirroring similar studies undertaken in the United States;
2. use the results of (1) to identify patterns and trends in spatial and temporal disaster declarations using spatial techniques and statistics;
3. examine for any obvious relationships between the frequency and distribution in space and time of the disaster declarations and the intensity of the El Niño/Southern Oscillation (ENSO) climate phenomena using appropriate statistical techniques;
4. determine whether the occurrence of disaster declarations are spatially clustered in NSW;
5. explore whether there are any associations between socio-economic disadvantage and ‘hot spot’ clusters of disaster declarations; and
6. to use the results of (1) to (5) to consider the implications for future disaster risk reduction planning and management by relevant State emergency management agencies.

We focus only on NSW since it is our home State, access to suitable disaster declaration data was readily available, we have a history of cooperation with the State emergency service authorities and understand their data needs and because NSW is the most populous State in Australia. Consequently, hazards and their associated disasters can be expected to affect significant proportions of the national population. Further, we are concerned with rapid onset disasters that are the remit of the Rural Fire Service (bushfires) and the State Emergency Service (floods and storms) to manage. We do not consider drought or heatwaves since they are not the responsibility of the State emergency service organizations to manage and we exclude tropical cyclones since none are known to have made landfall in NSW. We focus on the period from 2004 to 2014 since the dataset of disaster declarations is most complete for this period. Prior to this, only fragmented data on disaster declarations exists making temporal analyses unreliable.

## Results

### Total disaster declarations 2004–2014

**Figure 2:**
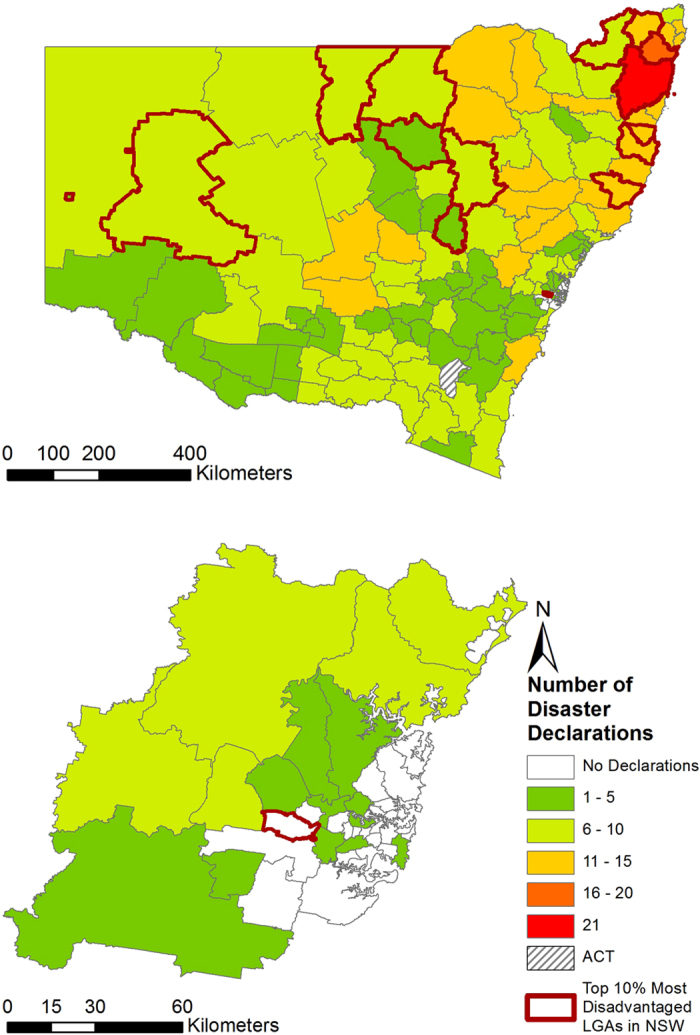
New South Wales and its Local Government Areas (LGAs). The map shows the number of times natural disasters were declared in each LGA. This map correlates with the disaster declarations listed in Table 1. Figure created using Esri ArcGIS 10.2 (https://esriaustralia.com.au/products-arcgis-software-102).

**Figure 3:**
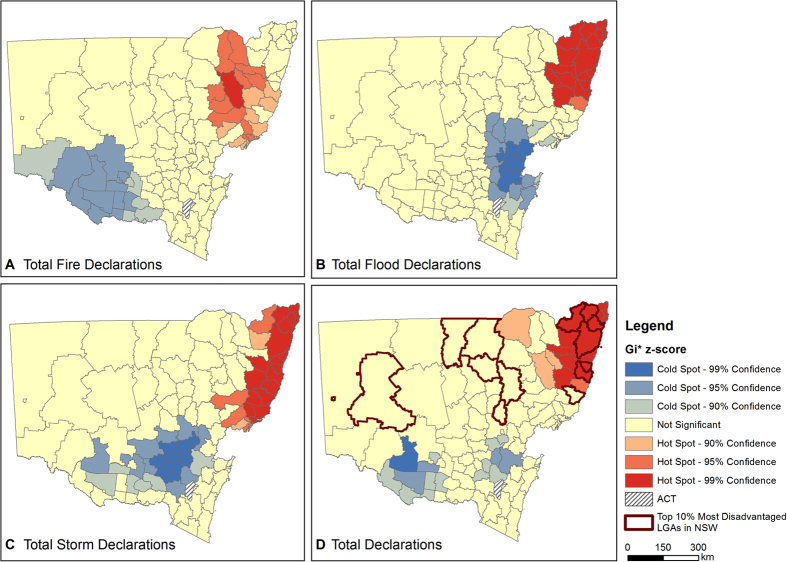
Spatial clusters of high and low disaster declaration between 2004 and 2014. High Getis-Ord 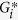 z-scores indicate more intense clustering of high declarations (hotspots), shown in red, and low z-scores show more intense clustering of low declarations (cold spots), shown in blue. (**A**) Total Bushfire Disaster Declarations; (**B**) Total Flood Disaster Declarations; (**C**) Total Storm Disaster Declarations; (**D**) Total Disaster Declarations (all types combined). The 10% most socio-economically disadvantaged LGAs in NSW are shown with dark boundaries. Figure created using Esri ArcGIS 10.2 (https://esriaustralia.com.au/products-arcgis-software-102).

Between 2004 and 2014, LGAs were included in natural disaster declarations 905 times – although the actual discrete number of separate events was 207. Table 1 provides the total number of LGAs experiencing disaster declarations by hazard type, by financial year. As indicated in Methods, this does not mean that there were 905 separate disasters. One event may have simultaneously affected four, or twenty or fifty LGAs. However, for the purposes of description and mapping which LGAs were affected, we note 905 declarations. The actual number of separate bushfire events was 108, of flood events was 44 and of storm events was 55. Figure 2 maps the disaster declarations noted in Table 1 and highlights the number of times disasters were declared in each NSW LGA. Twenty-seven LGAs experienced no disaster declarations. These are all located within the Greater Metropolitan Region. The five LGAs that experienced the most disaster declarations were Clarence Valley, Richmond Valley, Narrabri, Nambucca with 21, 16, 15 and 15 declarations respectively as well as Bellingen, Gwydir, Kyogle, Lismore and the Upper Hunter Shire which tied equal fifth place with 14 declarations each. Within the Greater Metropolitan Region, the Blue Mountains and Hawkesbury LGAs experienced the highest number of disaster declarations at 10 and 9 events, respectively. Results of the Global Moran’s *I* statistic allowed for rejection of the null hypothesis that disaster declarations were randomly distributed across NSW for all declaration types. The Getis-Ord 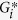 analysis by LGA showed a statistically significant cluster of high disaster declarations or ‘hotspot’ located in the northeast of the State and clusters of low disaster declarations or ‘cold spots’ located southwest of Sydney and in the central southern region of the State (Fig. 3D).

Broadly speaking, the number of disaster declarations increased each year through the time series and the most recorded in any year was 181 in 2012–2013. 2004–2005 was the year with the least disaster declarations. A marked increase in the number of disaster declarations occurred between 2008–2009 and 2009–2010 with the total number of disaster declarations per financial year remaining high since 2009–2010. Between 2004 and 2014 floods generated the greatest number of disaster declarations (*n* = 447) and storms the fewest (*n *= 255). For bushfires, 2012–2013 was the worst year with 119 disaster declarations. For floods, 2010–2011 was the worst year with 150 disaster declarations. For storms, 2012–2013 was the worst year with 62 disaster declarations. There were no years without a disaster declaration of some type although there were occasional years in which no bushfire or flood disaster declarations were made (Table 1).

### Bushfire declarations 2004–2014

**Figure 4:**
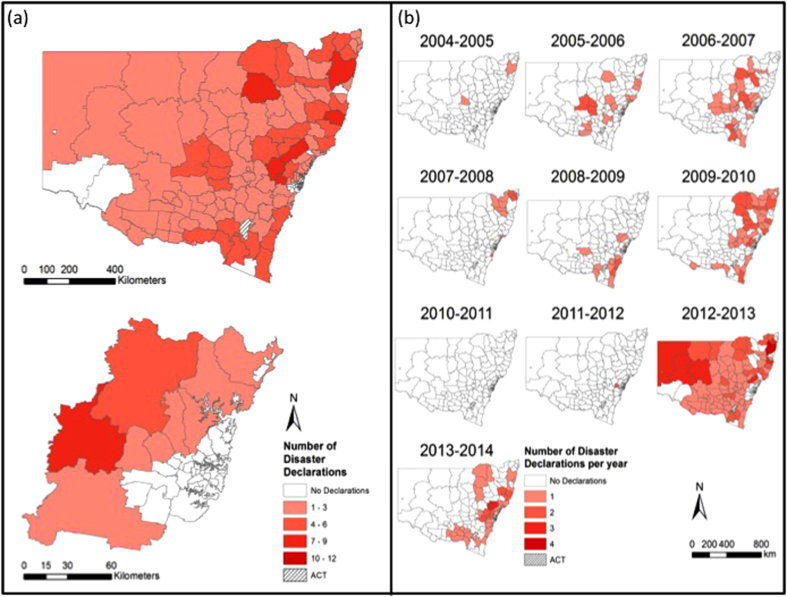
(**a**) Number of bushfire disaster declarations in each LGA; (**b**) Number of bushfire declarations in each LGA between 2004 and 2014. Figure created using Esri ArcGIS 10.2 (https://esriaustralia.com.au/products-arcgis-software-102).

In total, LGAs were included in bushfire disaster declarations on 319 occasions (Table 1). Very few LGAs were entirely free of a bushfire disaster declaration during this ten-year period, but those that were, were mostly confined to the southeast of the Greater Metropolitan Region and the far southwest of the State (Fig. 4a). The LGAs that experienced the most number of bushfire disaster declarations were largely confined to the northeast of the State (Fig. 4a). Singleton, the Clarence Valley and Narrabri experienced nine, eight and eight bushfire disaster declarations respectively. The Blue Mountains, Lithgow and Port-Macquarie Hasting rounded out the top five bushfire declared LGA’s with seven disaster declarations each. The Blue Mountains and Hawkesbury LGAs on the western fringe of the Sydney Metropolitan Region experienced the most bushfire disaster declarations at seven and five each (Fig. 4a). However, the Getis-Ord 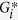 analyses determined a bushfire disaster declaration hotspot in the northeast of the State and a large cold spot in the central south of the State (Fig. 3A).

Figure 4b shows the time series of the number of bushfire disaster declarations in each LGA between 2004 and 2014. Financial year 2012–2013 was the worst year for bushfire disaster declarations across the State with a total of 119 declared disasters (Table 1, Fig. 4b). 2009–2010, 2013–2014 and 2006–2007 were also noteworthy for bushfire disaster declarations. Only 2010–2011 had no bushfire disaster declarations followed by 2004–2005 and 2011–2012 with only two each.

### Flood declarations 2004–2014

**Figure 5:**
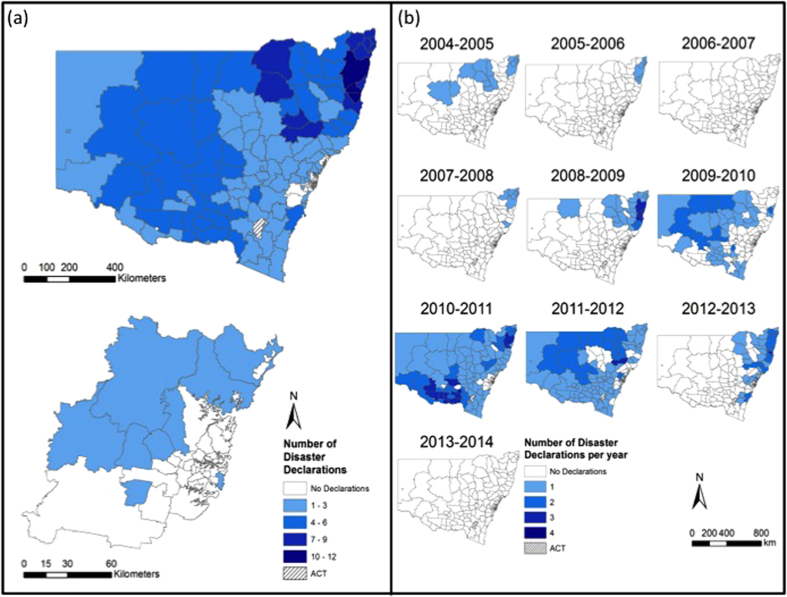
(**a**) Number of flood disaster declarations in each LGA; (**b**) Number of flood declarations in each LGA between 2004 and 2014. Figure created using Esri ArcGIS 10.2 (https://esriaustralia.com.au/products-arcgis-software-102).

In total, 447 flood disaster declarations were made (Table 1). Thirty-eight LGAs were entirely free of a flood disaster declaration during the study period and these were confined to the Greater Metropolitan Region (Fig. 5a). The LGAs that experienced the most number of flood disaster declarations were again largely restricted to the northeast of the State (Fig. 5a). The Clarence Valley, Bellingen and Nambucca experienced 12, 12 and 11 flood disaster declarations respectively. Ballina, Byron, Coffs Harbour, Lismore and the Richmond Valley followed with nine flood disaster declarations each. However, the Getis-Ord 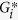 analyses show a clear flood disaster declaration hotspot in the northeast of the State and a disaster declaration coldspot in the area to the west-south-west of Sydney (Fig. 3B).

Figure 5b shows the time series of the number of flood disaster declarations in each LGA between 2004 and 2014. Financial year 2010–2011 was the worst year for flood disaster declarations across the State with a total of 150 disasters declared (Table 1, Fig. 5b). This was followed by 2011–2012 with 113 flood disaster declarations and 2012–2013 with 57 flood disaster declarations. Only 2006–2007 and 2013–2014 had no flood disaster declarations.

### Storm declarations 2004–2014

**Figure 6:**
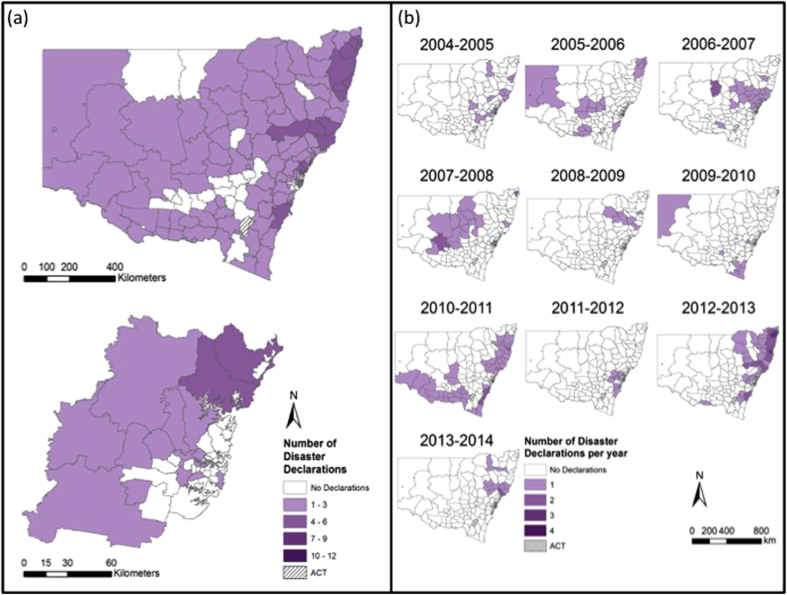
(**a**) Number of storm disaster declarations in each LGA; (**b**) Number of storm declarations in each LGA between 2004 and 2014. Figure created using Esri ArcGIS 10.2 (https://esriaustralia.com.au/products-arcgis-software-102).

The data show that 255 storm disaster declarations were made (Table 1). Forty-eight LGAs were entirely free of a storm disaster declaration during this ten-year period and these were confined to the south and eastern LGAs of the Greater Metropolitan Region, the State’s central regions and the central north (Fig. 6a). The LGAs that experienced the most number of storm disaster declarations were again largely restricted to the northeast and central coast of the State (Fig. 6a). Nambucca, Lismore, Greater Taree and the Shoalhaven each experienced six storm disaster declarations respectively. Ballina, Bellingen, Byron, Gloucester, the Great Lakes, Kiama and Wyong all experienced five storm disaster declarations. A hotspot of high storm disaster declarations along the northeast coast is clearly shown in the Getis-Ord 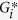 results (Fig. 3C). A local cluster of low storm disaster declarations (cold spot) is also evident in the central southeast region of the State.

Figure 6b shows the time series of the number of storm disaster declarations in each LGA between 2004 and 2014. Financial year 2012–2013 was the worst year for storm disaster declarations across the State with a total of 62 disasters declared (Table 1, Fig. 6b) – followed by 2010–2011 with 55 storm disaster declarations. No years were free from any storm disaster declarations although 2008–2009 was the year that recorded the fewest with just six (Table 1).

### Bushfire, flood and storm disaster declarations and the ENSO

A non-significant correlation between ENSO (as measured by the NINO3.4 index – see Methods) and bushfire disaster declarations of 0.19 (p = 0.27) was found, meaning that the current strength of ENSO is not a good predictor of the number of disaster declarations made. There is, however a difference between the two phases, with 45 disaster declarations made during active El Niño phases and 28 during La Niña phases. This means that during our study period, bushfire disaster declarations were more common during the El Niño, likely due to the hot and dry conditions that occur during this phase. This is in line with recent research that looked at the relationship between El Niño, extreme heat, and bushfires^16^.

The Spearman’s correlation between flood disaster declarations and the strength of ENSO was inconclusive but somewhat negative at −0.29 (p = 0.21), meaning that the strength of the ENSO phase is not a direct indicator of the total number of flood disaster declarations for a given month. However, disaster declarations for this hazard type were almost five times more common during La Niña phases (24 disaster declarations) compared to El Niño (five disaster declarations). Since La Niña phases are typically associated with higher than average rainfall, this result is not wholly unexpected. However, a five-fold increase in disaster declarations between the two active phases may provide some imperative to emergency services to prepare in advance.

Correlations with ENSO and storm disaster declarations were similar to flood declarations described above, also measured at −0.29 (p = 0.14). The difference between El Niño and La Niña phases is less impressive (nine and 17 disaster declarations, respectively). However, it still reflects the known relationships between ENSO and eastern Australian rainfall. Such results may still be useful for emergency services preparedness, as storm disaster declarations were almost twice as likely during La Niña phases compared to El Niño. Storms are also smaller in spatial scale compared to floods, and so are influenced by other meteorological and climatological mechanisms that operate on similar scales.

### Disaster declarations and socio-economic disadvantage

Fourteen rural LGAs in NSW have IRSAD scores in the lowest decile, representing the 10% most socio-economically disadvantaged communities in Australia (Table 2). Significantly, six of these LGAs (43%) are located within the total disaster declaration hotspot (99% confidence) shown in Fig. 3D (see Table 2). This shows a large proportion of the most socio-economically disadvantaged LGAs are experiencing significant numbers of disaster declarations.

## Discussion

The twenty-seven LGAs that experienced no disaster declarations were all located within the Greater Metropolitan Region, the part of NSW with the highest percentage of the overall State population. Consequently, during the study period of 2004 to 2014, the majority of the State’s population had no direct experience of hazards that led to disaster declarations. Although contested, it is well documented that prior experience is a strong predictor of increased individual and community awareness and where awareness is higher, affirmative risk mitigation behavior is generally higher^17^,^18^,^19^,^20^,^21^,^22^. The reverse is also the case where low experience and risk awareness lead to lower adaptive behaviours. A potential compounding issue is that emergency service organizations and their local response units and the emergency managers themselves, are not regularly responding to hazards occurring within their own jurisdictions so there might be a drop in readiness and capacity to respond in the future when hazards impact these LGAs^23^,^24^,^25^. Consequently, LGAs, their communities and local emergency management officials may be less prepared and more vulnerable to future hazards. Resources should be provided to emergency management officials and communities to assist them to prepare. Having said this, we acknowledge the possibility that emergency service managers, staff and volunteers in these LGAs have volunteered their services during emergencies and disasters elsewhere in the State and across Australia (and internationally), thus gaining insights and experiences that would be useful to their home jurisdictions. Further, it may be that LGAs in the Greater Metropolitan Region have more human and material resources available to them to prepare and respond to hazards. In either case, further research is needed to determine levels of preparedness in NSW LGAs.

Floods result in the most frequent disaster declarations. This would suggest that a focus on community education and engagement and allocation of resources to the State Emergency Service to support them to manage future floods would be especially helpful – although not at the expense of preparation for other hazard types. Such a focus on flood risk would be of value, particularly given research after the catastrophic floods in Queensland in 2011 demonstrated the need for education, risk communication and community knowledge in order to empower local communities to increase their flood resilience, with a surprising number of people indicating they had no idea they were at risk from floods^26^,^27^.

Whilst there were no significant Spearman’s correlations between bushfire, flood and storm disaster declarations and the respective phases of the ENSO, it is clear that bushfire and flood disaster declarations were more common during active El Niño or La Niña phases of the ENSO respectively. Several possibilities exist to explain this increased frequency of disaster declarations. First, it may be statistically true that more events occurred leading to disaster declarations. Second, it may be that the events that occurred were larger in geographic extent and therefore impacted more LGAs leading to greater numbers of disaster declarations. Third, the individual events may have been more intense leading to higher numbers of disaster declaration. Further research is needed to gain a better understanding of what exactly is happening and specifically, efforts to improve the number of data points and extend the time series would greatly help. What the results do suggest is that there is a complex relationship between meteorological conditions, early warning and a community’s preparedness and whether an event results in a disaster. This has implications for emergency service organizations as the ENSO shifts phase.

The overall pattern of disaster declaration occurrences in NSW was spatially clustered, as determined by the Global Moran’s *I* statistic. We are unable to explain why for each hazard type examined, there is a statistically significant ‘hot spot’ of disaster declarations located in the same northeast region of the State. It is likely that this has something to do with inherent locational vulnerability.

Whilst finer spatial scale analysis is required to establish the relationship between socio-economic disadvantage and disaster declarations, we consider the fact that 43% of the most disadvantaged LGAs are located within the disaster declaration hotspot to be significant from the perspective of disaster preparedness and response. This geographic overlap of disadvantage with frequent declaration of disaster presents challenges to emergency services, communities and governments that have to prepare for and respond to natural hazards. We do acknowledge the coarse scale of our spatial unit of analysis at the LGA level, noting that there will be heterogeneity within LGAs. Consequently, finer scale work is needed to examine local relationships at higher resolution scales such as ‘mesh block level’^28^,^29^. Having acknowledged this limitation, we note that for the hotspot in the northeast of the State, our data mirrors recent high-resolution (postcode level) analysis of disadvantage^30^.

Those interested in and responsible for the management of hazards and their associated disasters are concerned with a number of key questions. For example, what does Australia’s hazard landscape and disaster history look like? What types of hazard can we expect to affect Australia and what are their distributions, frequencies, magnitudes and return periods? What impacts and effects do they have on people, communities and the things we value? What technologies exist to forecast and predict events before they occur, and monitor and warn once an event has begun? What do individuals, families and communities understand about the risks associated with different hazards in Australia and what methods, tools, approaches and actions are available to enable them and our governments to reduce risk, increase resilience and safeguard us from disaster? Tied to this last question is what barriers and obstacles stand in the way of preventing individuals, communities and governments from taking actions that enhance resilience and reduce vulnerability? It is beyond the scope of the present study to attempt to tackle this list of questions (such a response is deserving of an entire publication in its own right). However, we note here that considerable research is on going to address these questions that builds upon a long scholarship in Australia focused on hazards and their accompanying disasters^31^,^32^,^33^,^34^,^35^,^36^,^37^,^38^,^39^,^40^. Such studies, as well as many others, have laid important foundations about the *what, where, when, how* and *why* of hazards and their accompanying disasters, against which more contemporary analyses and trends might be investigated and benchmarked. Further, they provide important insights and collectively track over time how well Australia is managing and mitigating the risks associated with hazards and their accompanying disasters.

Given the dynamic nature of our socio-ecological systems, this process never really ends. Consequently, there is a critical need to continuously assess what we know about hazards and the disasters they cause. This information can then be used to evaluate and modify if necessary, disaster risk reduction policies and practices. This study is a contribution to that effort.

## Methods

### Data Collection

Data about *Natural Disaster Declarations* in NSW were sourced from the NSW Ministry for Police and Emergency Services (MPES) website (2014). Any declaration that impacted multiple LGAs was counted once for each LGA in the declaration. That is, a single bushfire disaster (for example) might impact six LGAs resulting in us noting six bushfire disaster declarations (rather than one disaster declaration). Lord Howe Island, an autonomous island off the coast of NSW, was excluded, and declarations for LGAs amalgamated in the past ten years were attributed to the relevant LGA from the 2011 census. Declared disaster types were simplified in to one of three principal categories, ‘Bushfire’, ‘Flood’ and ‘Storm’. In relation to ‘Storm’ events, we collapsed a variety of event types in to the singular ‘Storm’ including ‘severe weather’, ‘storms and flooding’, ‘storms’, ‘storms, flooding and landslides’, ‘floods and storms’, ‘dust storms’, ‘wind storms’, ‘severe hailstorm’ and ‘severe hailstorm and wind storm’. Combined storm and flood events were counted once in each category, but only once for final disaster declaration totals. Totals by type and in aggregate form for all LGAs affected are shown in Table 2.

### Collation and analysis of relevant climate data

The El Niño/Southern Oscillation (ENSO) phenomenon is a known driver of climate over eastern Australia^41^. ENSO is quasi cyclical, oscillating every 2–7 years between El Niño (generally hot and dry conditions), neutral (average conditions) and La Niña (generally cool and wet conditions)^42^,^43^,^44^. It is most active during the Australian summertime. However, its influence on the climate can extend outside this season. In order to understand any relationships between ENSO and disaster declarations in NSW, the NINO3.4 index was used to represent the state of ENSO at the monthly timescale. We use the pre-calculated index from the National Oceanic and Atmospheric Administration (NOAA; http://www.cpc.noaa.gov/data/indices/). NINO3.4 measures sea surface temperature anomalies in the Central Equatorial Pacific (5°S–5°N, 170°W–120°W), where standardized values smaller than −0.5 represent La Niña conditions, and values greater than 0.5 represent El Niño conditions. Here we determine how many bushfire, flood and storm disaster declarations occur during active La Niña and El Niño phases of ENSO. We then calculated the nonparametric Spearman’s correlation coefficient to determine whether the strength of the state of ENSO can provide information about the number of disaster declarations per hazard type. Correlations are deemed significant at the 5% level.

### Mapping and geospatial analyses

Data about disaster declarations were linked with shapefiles of LGA boundaries from the Australian Bureau of Statistics’ 2011 Australian Standard Geographical Classification data^45^ in ArcGIS 10.2. The distribution of disaster declarations was mapped for four categories: bushfires, floods, storms and total disaster events, over the ten-year period from July 2004 until June 2014 (correlating with the Australian financial year). The number of disaster declarations for each hazard type was also mapped for each financial year to examine finer temporal scale variations.

The spatial distribution of disaster declarations in NSW was examined using the Global Moran’s *I* statistic^46^ to determine whether the overall pattern is clustered, dispersed or random. The null hypothesis was that disaster declarations were randomly distributed across NSW. The statistical significance of Global Moran’s *I* was tested using the z-score, the standardized difference between the observed and expected values, and p-value. The Getis-Ord 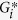, statistic, which measures the degree of spatial clustering of a local sample relative to the mean^47^, was then applied to test for the presence of local clusters. For both tests a binary weights case was applied using a fixed distance band weighting procedure. Appropriate lag distances were determined for each hazard type using incremental spatial autocorrelation. The lag distance or distance band defines the geographical area surrounding the LGA considered in the analyses and will reflect underlying processes influencing cluster patterns which may operate at different scales for each hazard type. LGAs within the Greater Sydney Area were removed from the cluster analysis due to differences in scale. The lag distance of 158 km was specified for floods, storms and total disasters and 223 km for bushfire. The false discovery rate (FDR) criterion was applied to adjust for multiple testing^48^. Positive 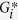 values indicate statistically significant spatial clustering of LGAs with high disaster declaration occurrences and negative values indicate spatial clustering of LGAs with low disaster declaration occurrences.

The Australian Bureau of Statistics’ 2011 Socio-economic Indexes for Areas (SEIFA) data was used to examine the spatial relationship between clusters of high disaster declarations and vulnerable communities. The SEIFA Index of relative socio-economic advantage and disadvantage (IRSAD) provided a proxy indicator for less resilient and more vulnerable communities following an established approach^49^. LGAs with an IRSAD decile of 1, indicating the 10% most disadvantaged communities in Australia, were identified and compared with spatial clusters of high disaster declaration occurrence.

## Additional Information

**How to cite this article**: Sewell, T. *et al*. Disaster declarations associated with bushfires, floods and storms in New South Wales, Australia between 2004 and 2014. *Sci. Rep.* **6**, 36369; doi: 10.1038/srep36369 (2016).

**Publisher’s note:** Springer Nature remains neutral with regard to jurisdictional claims in published maps and institutional affiliations.

## Figures and Tables

**Figure 1:**
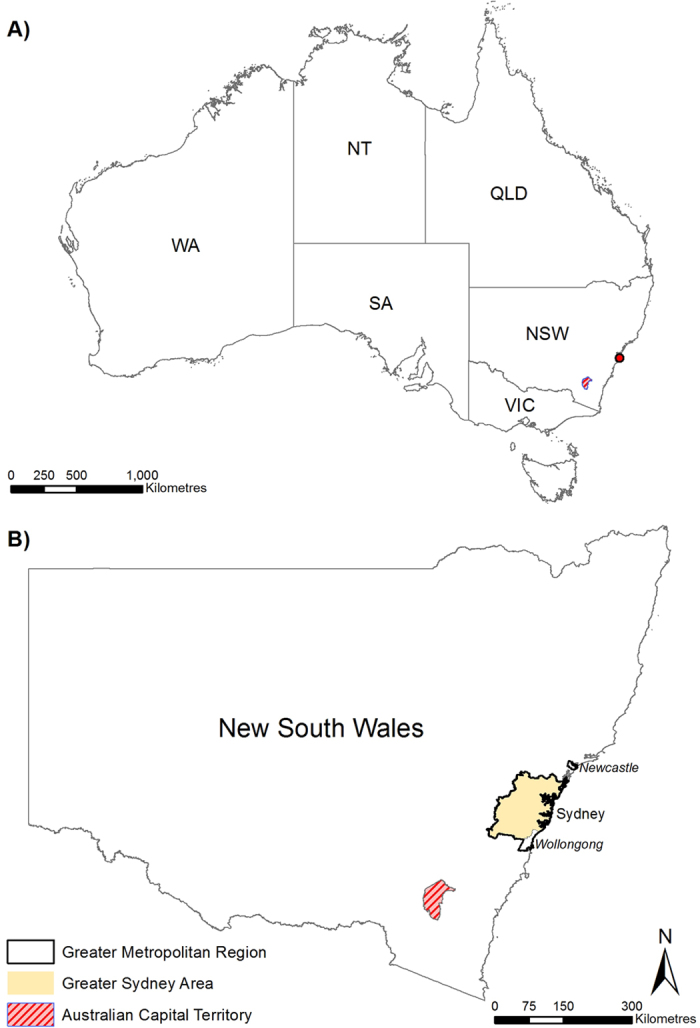
(**A**) Map of Australia. Red dot locates Sydney. The States include: New South Wales (NSW), Victoria (VIC), Tasmania (TAS), South Australia (SA), Western Australia (WA) and Queensland (QLD) as well as the two Territories: The Australian Capital Territory (ACT) and the Northern Territory (NT). (**B**) The State of New South Wales. The State is regularly affected by natural disasters associated with ‘hydro-meteorological’ hazards. See text for more details. The vast majority of the State’s population is located in the Greater Metropolitan Region between Newcastle and Wollongong. Figure created using Esri ArcGIS 10.2 (https://esriaustralia.com.au/products-arcgis-software-102).

**Table 1:** Total number of disaster declarations between 2004 and 2014 (by type).

Financial Year	Bushfires (actual separate events)	Number of LGAs included in bushfire disaster declarations	Floods (actual separate events)	Number of LGAs included in flood disaster declarations	Storms (actual separate events)	Number of LGAs included in storm disaster declarations	Total (actual separate events)	Total number of LGAs included in disaster declarations
2004–2005	2	2	2	16	5	21	9	39
2005–2006	11	23	2	9	10	27	23	54
2006–2007	17	34	0	0	6	24	23	58
2007–2008	6	15	2	9	7	24	15	47
2008–2009	8	15	8	38	3	6	19	54
2009–2010	24	56	13	55	6	10	43	121
2010–2011	0	0	8	150	5	55	13	160
2011–2012	2	2	5	113	3	11	10	123
2012–2013	23	119	4	57	6	62	33	181
2013–2014	15	53	0	0	4	15	19	68
**TOTAL**	**108**	**319**	**44**	**447**	**55**	**255**	**207**	**905**

**Table 2:** The 10% most disadvantaged LGAs in NSW (highlighted in Figs 2 and 3D).

Disadvantaged LGA	Within ‘hotspot’ shown in Fig. 3D
Brewarrina	No
Broken Hill	No
Central Darling	No
Clarence Valley	**YES**
Coonamble	No
Fairfield *	No
Greater Taree	No
Kempsey	**YES**
Kyogle	**YES**
Nambucca	**YES**
Richmond Valley	**YES**
Tenterfield	**YES**
Walgett	No
Warrumbungle Shire	No
Wellington	No

NOTE* Fairfield is the only LGA in the Sydney Metropolitan Region highlighted in Fig. 2 (lower).
